# Strength Training Improves Metabolic Health Markers in Older Individual Regardless of Training Frequency

**DOI:** 10.3389/fphys.2019.00032

**Published:** 2019-02-01

**Authors:** Johanna K. Ihalainen, Alistair Inglis, Tuomas Mäkinen, Robert U. Newton, Heikki Kainulainen, Heikki Kyröläinen, Simon Walker

**Affiliations:** ^1^Neuromuscular Research Center, Faculty of Sport and Health Sciences, University of Jyväskylä, Jyväskylä, Finland; ^2^Department of Health Sciences, Swedish Winter Sports Research Centre, Mid Sweden University, Östersund, Sweden; ^3^LIKES-Research Centre for Sport and Health Sciences, Jyväskylä, Finland; ^4^Centre for Exercise and Sports Science Research, Edith Cowan University, Joondalup, WA, Australia

**Keywords:** elderly, inflammation, monocyte chemoattractant protein-1, blood glucose, insulin, muscle mass, fat mass, exercise

## Abstract

The main purpose of the present study was to investigate the effect of frequency, thereby increasing training volume, of resistance training on body composition, inflammation markers, lipid and glycemic profile in healthy older individuals (age range 65–75 year). Ninety-two healthy participants were randomly assigned to one of four groups; performing strength training one- (EX1), two- (EX2), or three- (EX3) times-per-week and a non-training control (CON) group. Whole-body strength training was performed using 2–5 sets and 4–12 repetitions per exercise and 7–9 exercises per session. All training groups attended supervised resistance training for 6 months. Body composition was measured by dual X-ray absorptiometry and fasting blood samples were taken pre- and post-training. There were significant main effects of time for total fat mass (*F* = 28.12, *P* < 0.001) and abdominal fat mass (*F* = 20.72, *P* < 0.001). Pre- to post-study, statistically significant reductions in fat mass (Δ = -1.3 ± 1.4 kg, *P* < 0.001, *n* = 26) were observed in EX3. Pre- to post-study reductions in low density lipoprotein (LDL) concentration (Δ = -0.38 ± 0.44 mmol⋅L^-1^, *P* = 0.003, *n* = 19) were observed only in EX3, whereas a significant pre- to post-study increases in high density lipoprotein (HDL) concentration (0.14–0.19 mmol⋅L^-1^) were observed in all training groups. Most variables at baseline demonstrated a significant (negative) relationship when correlating baseline values with their change during the study including: Interleukin-6 (IL-6) (*r* = -0.583, *P* < 0.001), high-sensitivity c-reactive protein (hs-CRP) (*r* = -0.471, *P* < 0.001, and systolic blood pressure (*r* = -0.402, *P* = 0.003). The present study suggests that having more than two resistance training sessions in a week could be of benefit in the management of body composition and lipid profile. Nevertheless, interestingly, and importantly, those individuals with a higher baseline in systolic blood pressure, IL-6 and hs-CRP derived greatest benefit from the resistance training intervention, regardless of how many times-a-week they trained. Finally, the present study found no evidence that higher training frequency would induce greater benefit regarding inflammation markers or glycemic profile in healthy older adults.

## Introduction

Aging is associated with declines in levels of physical activity and once adults reach the age of 65, only 33% (from a peak of 55%) of the population fulfill the recommended amount of aerobic exercise. More worryingly, only 17% (from a peak of 30%) perform resistance training two or more times per week in United States (Schoenborn et al., 2013). Similar findings have been reported throughout the western world, including Finland (Bennie et al., 2017). As a consequence of this reduced level of physical activity, even healthy older individuals are more likely to experience adverse changes in their body composition (i.e., higher fat mass and lower muscle mass than younger individuals; Chumlea et al., 2002), as well as reduced aerobic fitness and strength (Häkkinen et al., 1998 et al., 1998; Sardeli et al., 2018; WHO, 2010; Ihalainen, 2018; Pedersen and Febbraio, 2008; Gleeson, 2011; Kraemer and Ratamess, 2005; Olson et al., 2007; Ibanez et al., 2010; Conceicao et al., 2013; Ribero et al., 2016; Williams et al., 2011; Marquies et al., 2009.; Andersson et al., 2011). These mal-adaptations in turn increase the likelihood of developing adverse metabolic conditions and low-grade inflammation. The metabolic syndrome, a cluster of conditions, including central obesity, dyslipidemia, hypertension and elevated fasting glucose, increases the risk of cardiovascular disease. Metabolic syndrome has been estimated to affect almost 50% of people over the age of 60 in United States (Aguilar et al., 2015). Resistance training (RT) in apparently healthy older adults has been shown to improve body composition by increasing muscle mass and decreasing fat mass (Sallinen et al., 2006; Walker et al., 2014), which should hypothetically lead to a reversal in the levels of metabolic risk factors and low-grade inflammation. Additionally, it has been suggested that repeated (beneficial) acute exercise responses would lead to decreased basal blood pressure (Halliwill, 2001) as well as low-grade inflammation (Petersen and Pedersen, 2005; Forti et al., 2016). However, results from intervention studies have been conflicting (e.g., Miller et al., 1994; Hagerman et al., 2000; Sillanpää et al., 2009; Tomeleri et al., 2018), and it seems that more widespread improvements from RT are found in those individuals with already diagnosed conditions, such as hypertension, type II diabetes, and obesity (e.g., Ibáñez et al., 2005; Sarsan et al., 2006; Strasser et al., 2010). Given that RT can provide a wide range of physical and psychological benefits (Liu and Latham, 2009), it is important to determine the potential advantage to prevent and treat age-related increases in markers of metabolic syndrome in the older, susceptible population.

The influence of RT on low-grade inflammation in older individuals has received less scientific attention. This is somewhat surprising since inflammation is a potential mechanism linking obesity and cardiometabolic risk. For example, favorable inflammatory status is positively associated with metabolic health (Phillips and Perry, 2013). Also, of great importance to an aging population, low-grade inflammation has been shown to be related to loss of muscle mass (Schaap et al., 2009) and muscle function (Schaap et al., 2006). Hence, considering its known interaction with adiposity, particularly abdominal adiposity (Strasser et al., 2012; Ihalainen et al., 2017a), the potency of RT for combating so-called inflammaging should be explored.

Metabolic and inflammatory changes induced by RT may be dependent on the specific characteristics of the exercise program (Calle and Fernandez, 2010; Lira et al., 2010; Nunes et al., 2016). In this regard, a recent study (Nunes et al., 2016) found that high-volume RT (six sets/exercise) was superior in the reduction in total cholesterol, LDL, waist-to-hip ratio and waist circumference compared to low-volume RT (three sets/exercise). Further Eklund et al. (2016) found that exercising more frequently (4 sessions per week) led to greater losses in fat mass than training twice a week, even when the training volume was matched. These greater abdominal fat losses led to greater reductions also in inflammation markers (Ihalainen et al., 2018), demonstrating a clear link between changes in fat mass and circulating levels of inflammation markers, even in normal-weight young subjects. In a recent meta-analysis on the effect of resistance training on inflammation markers in older adults, Sardeli et al. (2018) reported that only randomized controlled trials with a higher number of exercises (>8), higher weekly frequency (3 times/week) and durations longer than 12 weeks significantly reduced selected inflammation markers.

Consequently, it is logical to determine the potential influence of RT frequency, which modifies the overall training volume, over an extended period of time on markers of metabolic syndrome and low-grade inflammation in older adults. We hypothesized that greater training frequency would lead to greater reductions in body fat mass driving more favorable changes in markers of metabolic syndrome and low-grade inflammation levels in a group of healthy men and women over the age of 65 years.

## Materials and Methods

### Trial Design

The present study was a secondary analysis based on a parallel four-group randomized controlled trial “Get in Shape in the Team Research: Porukalla Kuntoon Tutkimus (PoKu)” (NCT02413112). Results for maximum strength, muscle hypertrophy, physical activity and functional capacity have been published previously (Turpela et al., 2017). All subjects were measured before (baseline) and after the 6-month intervention, which followed a 3-month preparatory training period (Walker et al., 2017). Three groups underwent supervised strength training at a specific training frequency (one-, two- or three-times-per-week), while one group acted as a non-training control group. The volume of RT followed the training frequency (EX1 = one-time-per-week, EX2 = two-times-per-week, EX3 = three-times-per-week) and, hence, volume was double in EX2 and triple in EX3 compared to EX1. The study was conducted according to the Declaration of Helsinki. Ethical approval was granted by the local ethics committee of the University of Jyväskylä, Finland (23.9.2013). Written informed consent was obtained from all subjects prior to inclusion.

### Participants

Eligible subjects for the study were community-dwelling 65–75 year old men and women not diagnosed with metabolic syndrome. Exclusion criteria were; (1) regular aerobic exercise (>180 min⋅week^-1^), (2) any previous strength training experience, (3) Body Mass Index (BMI) >37, (4) serious cardiovascular disease or lower limb injuries/disease that may lead to complications during exercise or affect the ability to perform testing and training, (5) use of walking aids, (6) use of medication that affect the neuromuscular or endocrine systems, (7) previous testosterone-altering treatment, and (8) smoking. Subjects that were taking any medication known to affect the variables within the present study were removed from the analyses.

While the subjects would be classed as not meeting the recommended physical activity levels (World Health Organization, 2010), they were active in low-intensity activities that are typical of a Nordic aged-population (e.g., berry-picking, forestry, gardening, walking/cycling etc.). All subjects were volunteers and did not receive any compensation for participation or travel expenses.

Subjects were recruited through prospective letters randomly sent to 2000 65–75 year olds living in the Jyväskylä region. Four hundred and fifty-four persons (23% response rate) registered to the study by submitting an online questionnaire, from which 148 apparently suitable candidates were invited to attend an information meeting. After the meeting, 116 persons provided written consent to the study and were subsequently invited to a physician’s examination. During the examination, 8 persons were deemed not eligible for the study on medical grounds. At this time, prior to randomization, one person withdrew due to lack of interest and another was no longer contactable. Consequently, 106 subjects were included to the study and randomly allocated to one of four groups; 3 intervention groups (EX1 = one-time-per-week, EX2 = two-times-per-week, EX3 = three-times-per-week) and 1 non-training/wait control group (CON). Thereafter, two women allocated to CON decided to withdraw from the study due to the results of randomization.

Baseline characteristics of the remaining subjects are shown in Table 1. There were no differences between groups for baseline data.

**Table 1:** General characteristics of the sample at baseline (mean ± SD).

	EX1 (*n* = 24)	EX2 (*n* = 24)	EX3 (*n* = 26)	CON (n = 20)
**General Characteristics**				
Sex (M/W)	11/13	10/14	12/14	11/9
Age (year)	69.8 ± 2.5	68.8 ± 2.9	69.5 ± 2.2	69.4 ± 2.2
Body mass (kg)	76.5 ± 14.5	80.6 ± 14.4	81.5 ± 14.7	75.1 ± 11.6
Height (m)	1.67 ± 8.7	1.68 ± 8.4	1.67 ± 9.3	1.68 ± 8.4
BMI (kg⋅m^-2^)	27.3 ± 3.3	28.5 ± 4.3	29.0 ± 4.1	26.4 ± 2.7
Fat mass (kg)	24.4 ± 6.6	26.8 ± 8.9	27.5 ± 8.3	22.4 ± 6.0
**Undiagnosed pathologies**				
TRIG ≥ 1.7 mmol⋅L^-1^	3	6	4	0
HDL < 1.03 or < 1.29 mmol⋅L^-1^	4	1	3	0
BP ≥ 130/85 mmHg	11	10	7	11
GLUC ≥ 6.1 mmol⋅L^-1^	3	5	6	1


*TRIG, triglycerides; HDL, high-density lipoprotein; BP, blood pressure; and GLUC, blood glucose.*

### Resistance Training Intervention

Detailed description of the study intervention has been reported previously (see supplementary material of Turpela et al., 2017; Walker et al., 2017). Briefly, after the initial 12 weeks of muscular endurance strength training two-times-per-week the intervention groups performed whole-body strength training either one- (EX1), two- (EX2) or three- (EX3) times-per-week for 6 months. This period was split into 2 mesocycles. The primary goal of the first 3-month mesocycle was to increase muscle mass and maximum strength. The primary goal of the second 3-month mesocycle was to increase maximum strength and muscle activation/power. Intensity for all upper and lower limb exercises was approximately 70–90% 1-RM with power training performed using 30–80% 1-RM loads.

All training sessions were supervised by experienced exercise instructors and each session was separated by at least 48 h recovery. All exercises were performed on commercially available weight-stack equipment (Precor Vitality Series^TM^, Precor Inc., United Kingdom) apart from several free-weight exercises in the last few weeks of training. All subjects were required to perform at least 1 set to concentric failure (with the exception of power training). All subjects were required to complete at least 90% of all allocated training sessions prior to testing. All subjects (intervention and control) recorded their daily leisure-time physical activity (external to the activity imposed within the study) in diaries prior to the study and throughout the 6-month period and 3-day diet diaries (including one weekend day) were collected. The recording of habitual physical activity external to the current intervention followed procedures of Waller et al. (2013). Subjects in the non-training control group were instructed to maintain their normal physical activity throughout the study period.

Due to the adherence requirements of the study (>90% adherence rate), 6 subjects were removed from the final analyses (1 from EX1, 2 from EX2, and 3 from EX3) based on non-compliance. The average weekly training attendance for the intervention groups throughout the study were; 1.0 ± 0.1 for EX1, 1.9 ± 0.1 for EX2 and 2.8 ± 0.2 for EX3. One (male) subject from EX1 was injured (back-pain) during strength testing and withdrew from the study. Reasons for other drop-outs were as follows; four (women) subjects withdrew due to illness unrelated to the study, and one man from CON could not be contacted at post-measurements.

### Primary Outcome Measurements

All measurements were performed following an overnight fast (12 h) with the subjects instructed to consume 0.5L of water in the morning prior to visiting the lab. Subjects were instructed to refrain from intensive exercise for at least 48 h prior to the tests. Testing took place between 7.00 and 9:00am, and each subject’s test time was fixed for the duration of the study (±30 min). The measurements were taken 6–7 days after the final training session of that period (i.e., after the last session of the 3-month primer and after the 6 month intervention). The measurements took place in May (after the 3-month primer) and December (after the 6-month intervention), 2015.

#### Body Composition

After determination of height by a fixed wall-mounted scale, participants underwent full body scanning by dual-energy x-ray absorptiometry (DXA) in minimal clothing (LUNAR Prodigy Advance with Encore software version 9.3, GE medical systems, United States). The legs were separated by a polystyrene block and secured by inelastic straps about the ankles. Arms were separated from the trunk by rice bags placed in the armpits and along the torso, palms were placed flat (prone) on the bed. Total body fat mass and lean mass were determined using software-generated analysis. Abdominal fat was taken as the software-generated android fat mass value. DXA measurement methods and validation have been reported by Salamone et al. (2000).

#### Blood Sampling Procedures and Analyses

Blood samples were taken from the antecubital vein using sterile techniques. Venous blood samples were collected into heparinized serum separator tubes (8.5 mL Venosafe SST 2 advance, Becton Dickinson and Co., vacutainer, Plymouth, United Kingdom), which stood at room temperature for 15 min before being centrifuged (5702R centrifuge, Eppendorf AG, Hamburg, Germany) for 10 min at 3,500 rpm. The serum was pipetted into 1.5 mL tubes and stored at -80°C until further analysis. Total and differential white blood cells (WBC), platelets, as well as hemoglobin and hematocrit were determined from EDTA-treated blood (Venosafe, Terumo, Belgium) with Sysmex KX-21N (TOA Medical Electronics Co., Ltd., Kobe, Japan). From the WBC; neutrophils, lymphocytes and mixed cells (monocytes, eosinophils, basophils and immature precursor cells) were differentiated and analyzed. Basic blood count (hemoglobin, hematocrit, white blood cell count etc.) was used to identify any possible illness (e.g., acute infection), which may have affected the inflammation data.

Serum samples were analyzed for glucose, insulin, glycated hemoglobin (HbA1c), interleukin-6 (IL-6), high-sensitivity C-reactive protein (hs-CRP), adiponectin, leptin, and cortisol using commercial chemiluminescence immunoassay techniques (Immulite 2000 XPi, Siemens Healthcare GmbH, Erlangen, Germany). Blood lipids and lipoproteins were also analyzed from serum (Konelab 20 XTi, Thermo Electron Co., Vantaa, Finland). Monocyte chemoattractant protein-1 (MCP-1) in serum samples was determined by enzyme-linked immunosorbent assay (ELISA) with commercial reagents (R&D Systems, Europe Ltd, Abingdon, United Kingdom).

The homeostatic model assessment (HOMA) was used to estimate insulin resistance (HOMA-IR) and %β–cell function (HOMA- β) from basal samples by the following equations:

$$Homa-IR=\frac{\text{fasting glucose concentration}\times \text{fasting insulin concentration}}{22.5}$$

$$Homa-\beta \left(\%\right)=\frac{\left(20xfasting\,\;insulin\,\;concentration\right)}{\left(fasting\,\;glu\,\cos e\,\;concentration-3.5\right)}$$

#### Blood Pressure Measurement

Upon completion of the basal blood sample, the subject’s blood pressure was taken using a calibrated and automated device (Omron M6W, Omron Healthcare Ltd, Hoofddorp, Netherlands). This ensured that the subjects had been sitting quietly for at least 10 min prior to the blood pressure test (approx. 5 min waiting and 5 min for blood sampling). The subjects placed their forearm on a table so that the elbow was at approximately 90° angle and the cuff was placed on the upper arm according to the manufacturer’s instructions. Three, separate measurements were taken. The lowest systolic value from the three measurements and the lowest diastolic value from the three measurements were used in subsequent analyses.

#### Oral Glucose Tolerance Test

Following the initial basal blood sample and blood pressure tests, subjects then consumed a standardized drink containing a 75g glucose load (GlucosePro, Comed Ltd, Espoo, Finland). While they waited for further blood samples, subjects underwent body composition tests in an adjacent room and otherwise sat in the waiting area of the lab. Blood samples related to the glucose tolerance test were obtained using the same methods described above at 60 and 120 min post-consumption.

### Statistical Methods

All statistics were performed using SPSS for Windows (IBM SPSS version 24.0; SPSS Inc., Chicago, IL). Conventional statistical methods were used to obtain means, standard deviation (SD) and Pearson’s product moment correlation coefficients. The Kolmogorov-Smirnov test was used to test normality and Levene’s test was used to analyze homogeneity of variance. Possible baseline between-group differences were assessed for 4 groups using a one-way analysis of variance (ANOVA). ANOVA with repeated measures was applied to test the intervention effects using a 4 group (EX1, EX2, EX3, CON) × 2 time (PRE, POST) design. Any significant main effects were assessed by Bonferroni *post hoc* tests for within-group differences. Significance was defined as *P* < 0.05.

## Results

### Body Composition and Blood Pressure

**FIGURE 1:**
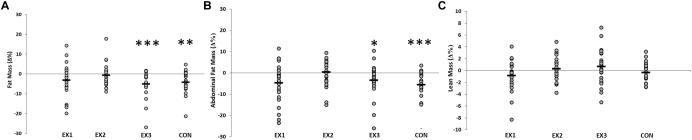
The effects of resistance training frequency on fat mass **(A)**, abdominal fat mass **(B)** and lean mass **(C)**. There were no significant time × group differences. Each subject (within each group) is represented by an O. The horizontal line represents the group mean (^∗^*p* < 0.05; ^∗∗^*p* < 0.01; ^∗∗∗^*p* < 0.001 versus baseline).

There were significant main effects of time for total fat mass (*F* = 28.12, *P* < 0.001) and abdominal fat mass (*F* = 20.72, *P* < 0.001) (Figure 1). Pre- to post-study, statistically significant reductions in total fat mass (Δ = -1.3 ± 1.4 kg, *P* < 0.001, *n* = 24) were observed in EX3 and in CON (Δ = -0.9 ± 1.2 kg, *P* = 0.004, *n* = 20). Significant reductions in abdominal fat mass were observed in EX1 (Δ = -0.1 ± 0.2 kg, *P* = 0.048, *n* = 25) and EX3 (Δ = -0.1 ± 0.2 kg, *P* = 0.033, *n* = 24), and in CON (Δ = -0.1 ± 0.1 kg, *P* = 0.020, *n* = 20). No within-group changes were observed in lean mass in any group during the present study (Table 2). There were no significant main effects for systolic or diastolic blood pressure.

**Table 2:** Effects of training frequency on body composition, cholesterol concentrations, markers of inflammation, blood glucose and blood pressure (mean ± SD).

	Within-group changes								Main Effects		
		
	EX1		EX2		EX3		CON		Time	Time × Group	Group
				
	pre-	post-	pre-	post-	pre-	post-	pre-	post-	(*P*-value)	(*P*-value)	(*P*-value)
Fat mass (kg)	25.4 ± 6.6	24.7 ± 7.1	26.8 ± 8.9	26.4 ± 8.2	27.5 ± 8.3	26.4 ± 8.4***	22.4 ± 6.0	21.5 ± 6.1**	<0.001	0.265	0.138
Fat-free mass (kg)	47.9 ± 12.1	47.5 ± 12.1	47.9 ± 10.4	47.9 ± 10.2	49.5 ± 10.1	49.6 ± 10.0	48.7 ± 10.2	48.5 ± 10.4	0.722	0.199	0.199
Abdominal fat mass (kg)	2.7 ± 0.7	2.6 @*0.8	2.8 ± 0.9	2.7 ± 0.9	3.2 ± 1.2	3.0 ± 1.2*	2.4 ± 0.6	2.2 ± 0.6***	0.016	0.289	0.049
GLU (mmol⋅L^-1^)	5.4 ± 0.5	5.4 ± 0.4	5.6 ± 0.6	5.9 ± 0.6*	5.6 ± 0.7	5.8 ± 0.6	5.4 ± 0.5	5.5 ± 0.4	0.016	0.121	0.076
INS (mmol⋅L^-1^)	51.2 ± 32.9	58.2 ± 38.1	39.7 ± 22.1	56.7 ± 28.3**	66.2 ± 37.3	73.2 ± 39.1	36.7 ± 20.4	49.5 ± 18.0**	<0.001	0.389	0.072
HOMA-IR	2.1 ± 1.4	2.1 ± 1.7	1.7 ± 1.0	2.5 ± 1.3***	2.6 ± 1.7	3.2 ± 1.7	1.6 ± 0.8	2.0 ± 0.8**	<0.001	0.460	0.026
HOMA-β (%)	88.2 ± 54.9	101.4 ± 62.1	63.1 ± 32.1	83.8 ± 30.4*	97.5 ± 51.4	108 ± 66.3	66.5 ± 36.9	83.1 ± 30.4**	0.001	0.865	0.095
HbA1c (mmol⋅mol^-1^)	35.3 ± 3.4	35.1 ± 3.6	35.0 ± 3.2	35.8 ± 2.9*	36.8 ± 5.9	36.0 ± 5.0	34.8 ± 3.1	34.8 ± 3.1	0.785	0.064	0.663
TC (mmol⋅L^-1^)	5.6 ± 1.1	5.8 ± 1.0	5.5 ± 0.6	5.9 ± 0.7**	5.6 ± 0.7	5.7 ± 0.7	5.9 ± 0.8	5.9 ± 0.7	0.006	0.150	0.829
HDL (mmol⋅L^-1^)	1.6 ± 0.5	1.7 ± 0.5*	1.6 ± 0.6	1.8 ± 0.3***	1.5 ± 0.4	1.6 ± 0.5**	1.9 ± 0.5	1.9 ± 0.5	<0.001	0.155	0.184
LDL (mmol⋅L^-1^)	3.8 ± 1.1	3.6 ± 0.9	3.8 ± 0.7	3.7 ± 0.7	3.9 ± 0.7	3.6 ± 0.9*	3.7 ± 0.6	3.7 ± 0.6	0.006	0.047	0.999
TRIG (mmol⋅L^-1^)	1.4 ± 0.6	1.3 ± 0.5	1.4 ± 0.5	1.3 ± 0.5	1.6 ± 1.0	1.6 ± 0.8	1.1 ± 0.3	1.1 ± 0.3	0.313	0.630	0.219
Cortisol (nmol⋅L^-1^)	360 ± 92	440 ± 91***	330 ± 95	420 ± 98**	310 ± 89	410 ± 80***	330 ± 97	430 ± 100***	<0.001	0.823	0.315
IL-6 (pg⋅ml^-1^)	1.8 ± 1.7	1.1 ± 0.8	1.5 ± 1.5	1.4 ± 1.7	1.2 ± 0.7	1.2 ± 1.3	1.1 ± 0.9	1.0 ± 1.3	0.546	0.290	0.370
hs-CRP (mg⋅ml^-1^)	2.6 ± 2.5	2.0 ± 1.9	1.7 ± 1.7	1.7 ± 1.5	2.3 ± 1.8	2.6 ± 2.1	1.5 ± 0.9	1.5 ± 1.3	0.817	0.210	0.142
Adiponectin (μg⋅ml^-1^)	9.2 ± 5.2	8.1 ± 4.3*	8.4 ± 4.3	7.2 ± 3.3*	7.8 ± 3.5	6.8 ± 2.8**	8.9 ± 3.1	8.3 ± 3.8	<0.001	0.823	0.510
Leptin (ng⋅ml^-1^)	53.3 ± 47.4	50.9 ± 41.3	49.7 ± 41.5	51.0 ± 37.2	43.4 ± 32.4	41.6 ± 28.6	35.2 ± 37.2	33.2 ± 31.4	0.426	0.818	0.374
MCP-1 (pg⋅ml^-1^)	365 ± 95	385 ± 88	387 ± 123	369 ± 138	374 ± 129	393 ± 154	415 ± 95	461 ± 112	0.110	0.257	0.408
SBP (mmHg)	140 ± 20	140 ± 20	155 ± 27	161 ± 32	142 ± 14	141 ± 18	149 ± 20	149 ± 17	0.506	0.601	0.052
DBP (mmHg)	77 ± 14	77 ± 14	80 ± 10	82 ± 10	76 ± 10	75 ± 14	79 ± 11	80 ± 11	0.679	0.890	0.315


*^∗^Significant within-group change from pre to post (^∗^*p* < 0.05; ^∗∗^*p* < 0.01; ^∗∗∗^*p* < 0.001 versus baseline). GLU, blood glucose; INS, insulin; HOME-IR, Homeostatic Model Assessment of Insulin Resistance; HOMA- β, homeostatic model assessment for β-cell function; HbA1c, Hemoglobin A1c; TC, total cholesterol; HDL, high-density lipoprotein; LDL, low-density lipoprotein; TRIG, triglycerides; IL-6, interleukin-6; hs-CRP, high-sensitive C-reactive protein; MCP-1, monocyte chemoattractant protein-1; SBP, systolic blood pressure; DBP, diastolic blood pressure.*

### Cholesterol Concentrations

Significant main effects for time were observed in total cholesterol (*F* = 7.97, *P* = 0.001) and in HDL concentration (Time: *F* = 37.56, *P* < 0.001). LDL concentration showed a significant main effect for time (*F* = 7.92, *P* = 0.006) and time × group (*F* = 2.80, *P* = 0.047) (Table 2). Pre- to post-study increases in HDL concentration were observed in all training groups: EX1 (Δ = 0.14 ± 0.20 mmol⋅L^-1^, *P* = 0.012, *n* = 18), EX2 (Δ = 0.19 ± 0.10 mmol⋅L^-1^, *P* < 0.001, *n* = 18) and EX3 (Δ = 0.15 ± 0.15 mmol⋅L^-1^, *P* = 0.001, *n* = 18). Pre- to post-study reductions in LDL concentration (Δ = -0.38 ± 0.44 mmol⋅L^-1^, *P* = 0.003, *n* = 19) were observed only in EX3.

### Markers of Inflammation

Cortisol demonstrated a significant main effect for time (*F* = 52.56, *P* < 0.001). There was also a significant main effect for time in adiponectin concentration (*F* = 24.76, *P* < 0.001) (Table 2). Statistically significant increases in cortisol were observed in EX1 (Δ = 74.8 ± 90.0nmol⋅L^-1^, *P* < 0.001, *n* = 24), EX2 (Δ = 96.6 ± 118.4nmol⋅L^-1^, *P* = 0.001, *n* = 25), EX3 (Δ = 95.1 ± 120.2 nmol⋅L^-1^, *P* = 0.001, *n* = 26), and CON (Δ = 105.2 ± 112.8 nmol⋅L^-1^, *P* = 0.004, *n* = 21). Statistically significant reductions in adiponectin were observed in all training groups: EX1 (Δ = -1.1 ± 2.2 μg⋅mL^-1^, *P* = 0.030, *n* = 24), EX2 (Δ = -1.2 ± 2.0 μg⋅mL^-1^, *P* = 0.011, *n* = 25) and EX3 (Δ = -1.0 ± 1.6 μg⋅mL^-1^, *P* = 0.004, *n* = 26). Significant changes or between-group differences in hs-CRP, MCP-1 or leptin were not observed.

### Blood Glucose

There were significant main effects of time for; basal glucose concentration (*F* = 6.084, *P* = 0.016), basal insulin concentration (*F* = 25,591, *P* < 0.001), HOMA-IR (*F* = 23.422, *P* < 0.001), and HOMA-β (*F* = 11.41, *P* = 0.01). HbA1C showed a trend for time × group interaction (*F* = 2.523, *P* = 0.064) (Table 2). Statistically significant increases in basal glucose concentrations (Δ = 0.3 ± 0.5 mmol⋅L^-1^, *P* = 0.038) were observed in EX2. Insulin concentration increased significantly in EX2 (Δ = 17.2 ± 15.5 mmol⋅L^-1^, *P* = 0.001), and CON (Δ = 11.0 ± 15.0 mmol⋅L^-1^, *P* = 0.005). These changes, in turn, led to significant changes in HOMA-IR in EX2 (Δ = 0.61 ± 0.85, *P* = 0.001) and CON (Δ = 0.47 ± 0.63, *P* = 0.008). HOMA-β also showed significant worsening in EX2 (Δ = 8.8 ± 37.8, *P* = 0.013), and CON (Δ = 16.6 ± 23.7, *P* = 0.007). Pre- to post-study increases in HbA1c were observed in EX2 (Δ = 2.5 ± 8.2 mmol⋅L^-1^, *P* = 0.028).

### Oral Glucose Tolerance

Typical responses to an oral glucose tolerance test were observed in glucose and insulin concentrations. Significant increases in both glucose and insulin occurred over the initial 60 min period, followed by decreased concentrations over the second 60 min period. However, there were no changes in glucose or insulin concentration at 60 or 120 min post-ingestion comparing pre- to post-study in any group.

### Relationships Between Baseline Values and Pre- to Post-study Changes

**FIGURE 2:**
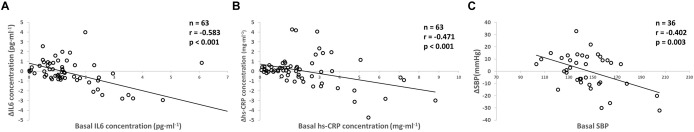
Relationship between the baseline interleukin-6 (IL-6, **A**), high-sensitive C-reactive protein (hs-CRP, **B**), and systolic blood pressure (SBP, **C**) with corresponding change during the study in training groups. Each participant’s data is marked by an O.

Most variables at baseline demonstrated a significant (negative) relationship when assessing their change during the study, when all training groups were pooled. Baseline IL-6 (*r* = -0.583, *P* < 0.001), hs-CRP (*r* = -0.471, *P* < 0.001), and systolic blood pressure (*r* = -0.402, *P* = 0.003) correlated significantly with their respective changes during the study (Figure 2).

### Undiagnosed Pathologies

The number of participants that had higher than recommended concentrations of triglycerides, blood pressure and blood glucose before the training intervention is presented in Table 1. After the intervention period, a total of four participants (EX1 = 1; EX3 = 3) with elevated fasting glucose concentration prior to the study achieved a normal range. Also, a total of five participants (EX1 = 2; EX2 = 1; EX3 = 2) reached the recommended HDL concentration and five participants (EX1 = 5) decreased to be below the recommended blood pressure values after the intervention.

### Habitual Physical Activity

Table 3 shows the physical activity from baseline and during the present study’s intervention period. There were no between-group differences in physical activity but CON significantly increased their activity from before the study to the present study period (*P* = 0.04).

**Table 3:** Habitual Physical Activity in intervention groups that trained one- (EX1), two- (EX2) or three- (EX3) times-per-week and in control group (CON).

	Baseline	Training intervention
EX1 (min⋅week^-1^)	113 ± 65	126 ± 125
EX2 (min⋅week^-1^)	116 ± 52	143 ± 130
EX3 (min⋅week^-1^)	87 ± 58	112 ± 109
CON (min⋅week^-1^)	116 ± 62	180 ± 76^∗^


*^∗^Significant within-group change from pre to post (^∗^*p* < 0.05 versus baseline).*

## Discussion

The purpose of the present study was to assess the effects of different frequencies of resistance training (RT) on markers of metabolic syndrome and low-grade inflammation in healthy older men and women. We expected that greater training frequency (and greater overall training volume load per week) would lead to greater reductions in body fat mass and greater increase in lean mass, which would then drive more favorable changes in markers of metabolic syndrome and low-grade inflammation levels in a group of healthy men and women over the age of 65 years. The main findings of this study showed that prolonged RT, at weekly frequencies of one-, two- or three-times-a-week, led to significant increases in HDL-cholesterol in all training groups. However, higher RT frequency might be needed to obtain significant reductions in LDL, total fat mass and abdominal fat mass. Nevertheless, higher loss in fat mass with more frequent training in the present study did not lead to greater improvements in markers of metabolic syndrome nor inflammation, contrary to our hypothesis. Last, it is noteworthy that the participants with the worst initial levels of metabolic syndrome and low-grade inflammation, particularly those with undiagnosed pathologies, improved the most due to training, regardless of the frequency.

Several studies have found RT to be effective for increasing muscle mass (Häkkinen et al., 1985; Narici et al., 1989) and reducing fat mass (Gettman et al., 1978; Hunter et al., 2004). However, training volume appears to be an important factor determining the training-induced magnitude of changes in body composition (Starkey et al., 1996; Nunes et al., 2016). Nunes et al. (2016) suggested that higher volume RT might be necessary to improve indicators of abdominal adiposity and lipid metabolism. Of the training groups in the present study, only training three-times-a-week led to significant reductions in total fat mass and abdominal fat mass of the healthy older men and women, which naturally has the highest total volume of training. Thus, the results of the present study are in-line with previous research in identifying that training volume has an important role in the exercise-induced loss in fat mass. However, there are also contradictory results. Ribeiro et al. (2015a) found no significant differences in changes in body composition, specifically fat mass and muscle mass, between older women performing a one-set or three-set RT protocol three-times-a-week. They concluded that in the initial state of training both volumes led to similar results. In another study by Ribeiro et al. (2015b), the effects of RT on body composition and health markers were affected by training status. Older women with no previous background in RT significantly lost fat mass whereas women with 24 weeks of RT experience did not lose fat mass. These collective findings highlight an important caveat in the present study. Since the subjects in the present study already had undergone 3 months of preparatory RT, they may have had a reduced potential for further loss of fat mass and only higher training volume (i.e., three-times-a-week) was sufficiently stimulating to cause further losses (Walker et al., 2017). This is perhaps also one reason for the somewhat unexpected lack of increased muscle mass in the present study.

Another notable aspect of the present study was that the magnitude of changes in fat mass in the present study was modest, -1.3 ± 1.4 kg in EX3 and -0.9 ± 1.2 kg in control group. Salamone et al. (2000) reported that DXA is an accurate method for measurement of fat mass in older individuals, however, should be acknowledged that precision of the repeated measurements expressed, as the percent coefficient of variation was 2.2% for fat mass in our laboratory. Therefore, it may be suggested that the observed intervention-induced change falls within the typical error of the measurement. However, due to the large n in all groups and prolonged intervention period, we find it improbable that a statistical error would explain the present findings. Indeed 21 out of 24 subjects in EX3, whereas 14 out of 20 subjects in CON reduced whole-body fat mass, supporting the idea that in particular EX3 demonstrated true change in fat mass.

The present study did not observe reductions in high-sensitive c-reactive protein (hs-CRP) or interleukin- 6 (IL-6) concentration. On the contrary, several studies have reported significant RT-induced reductions in inflammation markers in older adults (Tomeleri et al., 2018). Training induced changes in inflammation markers are more likely in the initial phase of resistance training (Ihalainen et al., 2017a). The reason for the contradictory results could be related to the fact that the participants in the present study did 12 weeks of resistance training prior this study. It is noteworthy that similarly to the study by Tomeleri et al. (2018), the subjects in the present study were healthy and undiagnosed for pathologies. Therefore, it is difficult to determine whether the pre-existing health status of the subjects could influence these comparisons.

Another reason for the contradictory results could be related to the lack of training-induced gain in muscle mass in any group in the present study. Sardeli et al.’s (2018) recent meta-analysis reported that randomized controlled trials failing to increase muscle mass did not reduce hs-CRP concentration, whereas randomized controlled trials that increased muscle mass also decreased hs-CRP. Sardeli et al. (2018) stated that changes in body composition determine the anti-inflammatory effects of RT. The authors suggested that the physiological mechanisms explaining beneficial effects of increased muscle mass on inflammation could be that RT increases energy expenditure and insulin sensitivity (Calle and Fernandez, 2010) and that higher muscle mass has more potential to produce anti-inflammatory myokines (Pedersen and Febbraio, 2008; Ihalainen et al., 2017b). Another mechanism that has been suggested to be responsible for the anti-inflammatory effect of RT has been the reduction of fat mass (Gleeson et al., 2011). Interestingly, despite the significant but modest beneficial loss in fat mass in EX3 and CON, the present study did not detect further significant beneficial effects of RT on inflammation markers. Therefore, there may be a threshold for body composition changes that influence inflammation status, prior to which no changes would be expected. To our knowledge, such a threshold (either fat mass reduction or muscle mass increase or both) has not yet been identified.

Interestingly, in the present study, adiponectin concentration was reduced in all training groups. Adiponectin, also known as Acrp30, apM1, GBP28 or AdipoQ, is a complex biomarker and there is currently no consensus regarding whether high concentrations represent improved or poorer health status, not to mention whether adiponectin itself plays a role in metabolic health (Waragai et al., 2018). Since adiponectin has been shown to have major anti-diabetic, anti-atherogenic and anti-inflammatory properties, it seems logical that a higher concentration is beneficial. Furthermore, higher adiponectin concentration is negatively correlated with fat mass, central fat distribution and fasting insulin (Su et al., 2011). However, higher adiponectin concentration is also associated with increased all-cause mortality and the association has been suggested to be strengthened when high levels of adiponectin are combined with low body mass index (Choi et al., 2015; Menzaghi and Trischitta, 2018). In the aging population, adiponectin concentration has been shown to increase and is found at relatively high concentrations 5–10 μg⋅mL^-1^. in the circulation even in healthy older humans.

Resting levels of cortisol are thought to reflect general physiological stress with possible changes regulating tissue homeostasis and protein metabolism (Kraemer and Ratamess, 2005). Basal cortisol concentrations following strength training in older individuals have typically remained unchanged (Häkkinen et al., 2000; Ibáñez et al., 2008). Contrary to previous studies, a significant increase in cortisol concentration was observed in all training groups, as well as in the control group. Since the changes were similar in all groups, the change is perhaps not due to the strength training intervention of the present study. One possible explanation could be that seasonal variations in cortisol concentration observed in high latitudes (Walker et al., 1997) have led to the present findings. Nevertheless, the potency of cortisol on tissue such as muscle is unable to be determined from tracking its concentration. For example, at rest trained individuals’ tissues are less sensitive to glucocorticoid action than non-trained individuals (Duclos et al., 1999). Hence, interpretations into cortisol concentrations should be made with caution.

There are few randomized controlled trials that have investigated exercise effects on adiponectin. Some have demonstrated a significant increase in adiponectin concentration both after resistance (Olson et al., 2007) and aerobic training (Mujumdar et al., 2011), contrasting the present study’s results. However, the lowering of adiponectin concentration in the present study is in-line with the results from Ibáñez et al. (2010). These authors reported that weight loss through diet only led to significant increase in adiponectin concentration whereas, a 16-week combined progressive RT and weight-loss diet led to significant decreases in circulating adiponectin that was accompanied by significant improvements in different cardiovascular risk factors. Also, Ihalainen et al. (2017a) reported a significant inverse relationship between change in concentration of circulating adiponectin and change in total lean mass after 12 weeks of RT in untrained young men. One explanation for these apparently conflicting findings on the effects of RT on adiponectin concentration could be changes in adiponectin multi-dimer ratio (Blüher et al., 2006; O’Leary et al., 2007) or changes in adiponectin receptor expression in skeletal muscle (O’Leary et al., 2007). However, the mechanisms underlying these training-induced changes and indeed the possible implications for health remains unresolved.

Regarding cholesterol, the results of the present study demonstrate a favorable response of HDL in all training groups. In addition, RT three-times-a-week led to a significant reduction in LDL. Non-optimal lipoprotein levels, high LDL and low HDL cholesterol, are a major risk for coronary heart disease. Furthermore LDL increases with advancing age. Regular exercise, especially aerobic exercise, has been proposed to be a potent approach for obtaining a healthy lipid profile. RT has also been shown to have potential to modify lipoprotein levels (Ibáñez et al., 2010). All present changes in concentrations of cholesterol and its fragments could be considered positive, and are in-line with previous studies (Williams et al., 2011; Conceição et al., 2013; Ribeiro et al., 2016). However, several intervention studies have not observed any effects of RT on lipoproteins (Marques et al., 2009; Orsatti et al., 2014). Ibáñez et al. (2010) suggested that the lack of significant lipoprotein-lipid changes with RT could be due to subjects already having moderate to low levels of lipoproteins at baseline as was the case with most of our subjects. Furthermore, individuals with non-pathological lipid profiles might require greater exercise stimuli and energy expenditure leading to changes in body composition. The results of the present study support this hypothesis as LDL reduced only in the group that trained three-times-a-week and reduced total fat and abdominal fat mass. Controversially, in the present study, an increase in HDL was observed even in a group that trained only once per week. Kodama et al. (2007) concluded in their meta-analysis that there appears to exist a minimum exercise volume of 120 min of aerobic training per week for a significant increase in HDL. If this observation were confirmed in future studies, then it signifies a potent HDL response to RT, which may be recommendable for those individuals at the borderline to become clinical populations to achieve positive cholesterol changes.

It has been suggested that exercise is medicine for the vast majority, if not to all. However, it has been shown that there are significant individual differences in the exercise-induced changes in performance as well as in selected health benefits (Mann et al., 2014; Ahtiainen et al., 2016) The present study showed that exercise was more effective for the corresponding health markers in subjects with initially high inflammation marker concentration (hs-CRP and IL-6) and systolic blood pressure. Previous studies have shown that individuals who benefit most from exercise regimens are the ones with previously low HDL-cholesterol levels, greater abdominal adiposity and elevated serum triglyceride levels (Couillard et al., 2001; Bouchard and Rankinen, 2001). Overall, our findings enforce the perception that suitable strength training interventions should be targeted to people with the poorest health parameters concerning both body composition and inflammation profile.

The present study has several limitations that should be discussed. The fact that the control group improved body composition could be explained by the increase in the habitual endurance-type physical activity during the intervention period (Walker et al., 2017). This is an unfortunate and unforeseen weakness of the present study, whereby control subjects were instructed to maintain their normal physical activity levels but did not comply. This finding does highlight the need to track habitual physical activity levels during intervention studies, but perhaps using diaries (where the subjects were not blind to their activity level) was not a suitable method for these purposes. Secondly, the design of the study may have limited the effectiveness of the intervention regarding increasing muscle mass. Specifically, there was a 3-month preparatory training period that already induced muscle hypertrophy compared to baseline in these individuals (Walker et al., 2017). Furthermore, older adults are purportedly “anabolically resistant” requiring greater and more frequent systematic protein ingestion compared to young individuals in order to achieve muscle hypertrophy (Morton et al., 2018). Hence, in order to invoke further muscle hypertrophy, to distinguish the potency of higher training frequency, and establish whether increased muscle mass leads to improved low-grade inflammation and glycemic profile, an exercise + nutritional intervention may have been a better study design. Despite these limitations, this study provides support for the effectiveness of progressive RT on the metabolic health in older men and women.

## Conclusion

In conclusion, the present study suggests that a higher number of RT sessions per week could be of benefit in the management of body composition and lipid profile. Interestingly, and importantly, the study observed that those individuals with a higher baseline systolic blood pressure, triglyceride and hs-CRP concentrations derived greatest benefit from the RT intervention, regardless of how many times-a-week they trained. Finally, the present study found no evidence that higher training frequency would induce greater benefit regarding inflammation markers or glycemic profile in healthy older adults. From a practical point of view, our findings suggest that suitable strength training interventions should be especially targeted to people with poorer body composition and metabolic profile.

## Author Contributions

SW, AI, HKa, RN, and HKy planned and initiated the study. AI, SW, and TM were responsible for the data collection. JI and SW were responsible for carrying out analyses and manuscript writing. JI, AI, and SW interpreted the results of research. All authors drafted, edited, critically revised the paper, and approved the final version of the manuscript.

## Conflict of Interest Statement

The authors declare that the research was conducted in the absence of any commercial or financial relationships that could be construed as a potential conflict of interest.
